# A Dual Infection Pseudorabies Virus Conditional Reporter Approach to Identify Projections to Collateralized Neurons in Complex Neural Circuits

**DOI:** 10.1371/journal.pone.0021141

**Published:** 2011-06-16

**Authors:** J. Patrick Card, Oren Kobiler, Ethan B. Ludmir, Vedant Desai, Alan F. Sved, Lynn W. Enquist

**Affiliations:** 1 Department of Neuroscience, University of Pittsburgh, Pittsburgh, Pennsylvania, United States of America; 2 Department of Molecular Biology and the Princeton Neuroscience Institute, Princeton University, Princeton, New Jersey, United States of America; French National Centre for Scientific Research, France

## Abstract

Replication and transneuronal transport of pseudorabies virus (PRV) are widely used to define the organization of neural circuits in rodent brain. Here we report a dual infection approach that highlights connections to neurons that collateralize within complex networks. The method combines Cre recombinase (Cre) expression from a PRV recombinant (PRV-267) and Cre-dependent reporter gene expression from a second infecting strain of PRV (PRV-263). PRV-267 expresses both Cre and a monomeric red fluorescent protein (mRFP) fused to viral capsid protein VP26 (VP26-mRFP) that accumulates in infected cell nuclei. PRV-263 carries a Brainbow cassette and expresses a red (dTomato) reporter that fills the cytoplasm. However, in the presence of Cre, the dTomato gene is recombined from the cassette, eliminating expression of the red reporter and liberating expression of either yellow (EYFP) or cyan (mCerulean) cytoplasmic reporters. We conducted proof-of-principle experiments using a well-characterized model in which separate injection of recombinant viruses into the left and right kidneys produces infection of neurons in the renal preautonomic network. Neurons dedicated to one kidney expressed the unique reporters characteristic of PRV-263 (cytoplasmic dTomato) or PRV-267 (nuclear VP26-mRFP). Dual infected neurons expressed VP26-mRFP and the cyan or yellow cytoplasmic reporters activated by Cre-mediated recombination of the Brainbow cassette. Differential expression of cyan or yellow reporters in neurons lacking VP26-mRFP provided a unique marker of neurons synaptically connected to dual infected neurons, a synaptic relationship that cannot be distinguished using other dual infection tracing approaches. These data demonstrate Cre-enabled conditional reporter expression in polysynaptic circuits that permits the identification of collateralized neurons and their presynaptic partners.

## Introduction

Neurotropic viruses represent popular and powerful tools for defining the identity and organization of synaptically connected neurons [1], [2], [3], [4], [5], [6], [7], [8], [9]. The method exploits the tropism of these viruses for neurons and their tendency to replicate and spread from neuron-to-neuron via the intimate synaptic contacts through which neurons communicate. Pseudorabies virus (PRV), a DNA swine alpha herpesvirus, is one of the most widely applied viruses for polysynaptic circuit analysis in the rodent nervous system. The extensive use of PRV in such studies is related to the availability of strains of reduced virulence that are transported selectively in the retrograde direction through neural circuits and also express unique reporter proteins (*e.g.*, fluorescent proteins).

Restricting PRV replication to defined populations of neurons through cell-specific gene expression is a recent advance of viral transneuronal tracing technology [10]. In an early report, DeFalco and colleagues exploited Cre-Lox site-specific recombination [11] of the viral genome to restrict PRV replication to phenotypically defined neurons and synaptically connected circuits [12]. Replication of the virus constructed for that study (PRV-2001) requires Cre-mediated recombination of the viral genome to remove a floxed stop cassette that prevents transcription of a thymidine kinase gene essential for viral replication in non-mitotic cells. Once the stop cassette is removed, the virus is permanently replication competent and passes transneuronally to infect synaptically linked neurons. This approach has subsequently been employed to define neural circuits synaptically linked to LHRH- [13], [14], [15] and serotonin- [16] containing neurons.

The aforementioned conditional approach is limited by the requirement that Cre be present in first-order neurons infected by PRV-2001. The approach cannot be used if the goal is to define synaptic connections specific to a population of Cre-expressing neurons embedded within a larger circuit, while maintaining target cell specificity of the circuit (*e.g.*, Cre-expressing neurons separated from the injection site by more than one synapse). The recent development of a replication competent PRV recombinant that changes the profile of reporter gene expression when exposed to Cre has circumvented this problem [17], [18]. The conditional recombinant, PRV-263, carries the Brainbow 1.0L cassette developed by Lichtman and colleagues [19] and expresses a red dTomato cytoplasmic reporter unless it has been exposed to Cre. In the presence of Cre, the red reporter gene is removed and either the cyan (mCerulean) or yellow (EYFP) reporter is expressed. It is important to emphasize that each virus can express only a single reporter (before or after Cre-recombination) but that infected neurons can replicate more than one viral genome, resulting in a mixed reporter phenotype of some infected neurons. We recently documented the utility of this approach for circuit analysis by combining PRV-263 infection with lentivirus-mediated expression of Cre in phenotypically-defined, anatomically localized, and projection-specific populations of neurons [18].

In this report we document a dual infection transneuronal tracing approach to identify neurons synaptically linked to collateralized neurons within complex networks. The method takes advantage of the Cre-conditional reporter expression of PRV-263 and a new strain of PRV (PRV-267) that expresses both Cre and an mRFP-capsid fusion protein (VP26-mRFP). Injection of PRV-267 and PRV-263 into separate kidneys using a well-characterized dual infection paradigm [20] produced unique markers of collateralized neurons synaptically linked to both kidneys (nuclear mRFP and conditional fluorescent reporter expression from the Brainbow cassette) as well as neurons infected by replication and transneuronal passage of progeny virus from those neurons (only Brainbow reporters). This approach expands the utility of dual infection viral transneuronal tracing paradigms by providing a means of distinguishing collateralized neurons within complex networks from the neurons that are antecedent to them presynaptically.

## Materials and Methods

### Ethics Statement

All experimental procedures involving animals conformed to regulations stipulated in the *NIH Guide for the Care and Use of Laboratory Animals* and were approved by the University of Pittsburgh IACUC (protocol number: 0909666), Recombinant DNA Committee (reference number: 112-09), and Division of Environmental Health and Safety (protocol number: 0909666). The *in vitro* experiments used to construct and characterize PRV-267 were conducted at Princeton University and approved by the Recombinant DNA Technology Committee (MUA # 912).

### Animals

Adult male rats (Harlan Sprague-Dawley) weighing 250 to 320 grams at the time of viral injection were used for *in vivo* experiments conducted in a Biosafety Level 2 certified laboratory. Animals were single housed and lived within this facility after virus injection. Photoperiod (12 hours light; light on at 0700) and temperature (22–25°C) were standardized and animals had free access to food and water.

### PRV Recombinants

**Figure 1:**
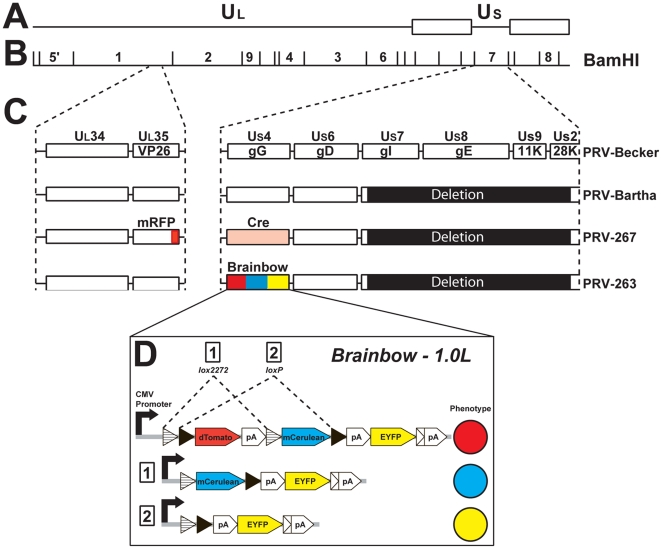
PRV Genome. The genomic organization of PRV-Becker, PRV-Bartha and the recombinants prepared for this study are illustrated. The basic organization of the PRV genome is illustrated at the top of the figure (A). Viral DNA contains unique long (Ul) and unique short (Us) segments flanked by internal and terminal repeat sequences. The BamH1 restriction map of PRV (B) illustrates the location of the portions of the viral genome engineered to express transgenes in PRV-263 and PRV-267. These regions of the restriction map are expanded in C to illustrate the location of the genes in segments 1 and 7 (C). Boxes represent individual genes, with the formal name indicated above the box and the common name, where appropriate, indicated within each box. The recombinants prepared for this analysis are derived from the PRV-Bartha genome, which contains a large deletion in the Us segment. The genes eliminated by this deletion reduce virulence and restrict viral transport through circuits to the retrograde direction. The Brainbow cassette and Cre were inserted into the gG (Us4) locus to create PRV-263 and PRV-267, respectively. The organization of the Brainbow 1.0L cassette is illustrated in section D of the figure. Paired loxP and lox2272 sites are positioned within the cassette such that recombination at loxP sites eliminates the dTomato and mCerulean genes to liberate expression of EYFP and recombination at lox2272 sites eliminates the red reporter gene to liberate expression of mCerulean. It is important to note that Cre only cuts at like pairs (*e.g.*, loxP∶loxP or lox2272∶lox2272) and that the cassette (intact or recombined) will only express one reporter. PRV-267 also carries mRFP as part of a fusion gene at the VP26 (Ul35) locus to produce a unique marker of the surface capsid protein VP26. Construction of PRV-267 is described in the Materials and Methods. Construction of PRV-263 has been reported [17].

The genomic organization of the recombinants and related strains of PRV are illustrated in Figure 1. The preparation of PRV-263, a PRV-Bartha recombinant carrying the Brainbow 1.0L cassette in the *US4* (gG) locus (Figure 1C), has been previously described [17]. PRV-267, a PRV-Bartha recombinant expressing Cre-recombinase and a red fluorescent protein-tagged VP26 capsid protein, is a new virus constructed for this study. Preparation of a Cre-containing plasmid (pEL2) and construction of the virus are described below.

To construct the pEL2 plasmid, the Cre-recombinase coding region, including an N-terminal nuclear localization signal and 133-base-pair synthetic intron was amplified by PCR from pBecker3 (a self-recombining bacterial artificial chromosome) as previously described [21]. Two PCR primers were designed with a *Kpn*I restriction site in the sequence homologous to the 5′ Cre open reading frame (ORF) (5′-GGGGTACCATGCCCAAGAAGAAGAGGAAG) and an *Xba*I site in the sequence complementary to the 3′ Cre ORF (5′-GCTCTAGACATATCGCCATCTT CCAGCAG). The eGFP ORF of pEGFP-N1 was removed through *Kpn*I/*Xba*I digestion, followed by ligation with the amplified Cre ORF. The resulting plasmid, pEL2, contained the Cre-recombinase coding region under immediate-early human cytomegalovirus (hCMV) promoter control. Restriction fragment analysis and nucleotide sequencing verified the structure of the plasmid.

PRV-267 was constructed as follows. We first constructed PRV-266, a PRV-Bartha strain expressing mRFP-VP26 and diffusible eGFP, through co-infection of porcine kidney epithelial (PK-15) cells with PRV-152 (a PRV-Bartha strain encoding a diffusible eGFP under an immediate-early hCMV promoter from the *US4* locus, [22]) and PRV-756 (a mRFP-VP26 fusion protein, [23]). VP26 is a surface capsid protein encoded by the UL35 gene and the fusion protein incorporating mRFP labels viral capsids intensely, thereby providing a unique marker of neurons replicating PRV-267. We selected viral recombinants expressing both mRFP-VP26 and diffusible eGFP; sequential rounds of plaque purification of this virus, PRV-266, were then performed. We then co-transfected PRV-266 nucleocapsid DNA (purified as previously described, [24]) with linearized pEL2 in PK15 cells. Homologous recombination resulted in PRV-267. We selected viral recombinants expressing mRFP-VP26, but not eGFP, to distinguish PRV-267 from PRV-266 and conducted sequential rounds of plaque purification to isolate PRV-267.

### Experimental design

**Figure 2:**
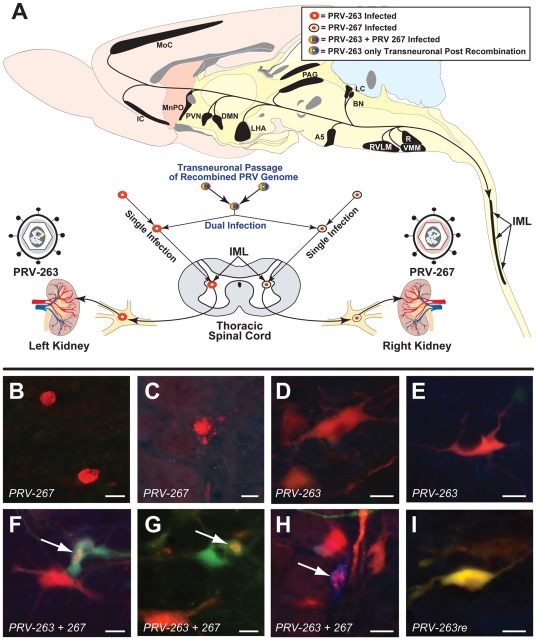
Experimental Paradigm and Neuronal Phenotypes. The experimental paradigm used in this analysis (A) and the reporter phenotypes of neurons infected with PRV-263 and PRV-267, either alone or in combination (B–I), are illustrated. Each animal received separate injections of PRV-263 and PRV-267 into the left or right kidney (A). Prior studies have demonstrated that PRV-Bartha recombinants are transported from the kidney to infect postganglionic neurons in the inferior mesenteric ganglion. Subsequent replication and transneuronal passage of virus infects sympathetic preganglionic neurons in the IML and neurons of the renal preautonomic network. The preautonomic network linked to each kidney is largely lateralized but also contains neurons that collateralize to innervate circuits linked to each kidney. Neurons infected with both recombinants express unique reporters (cyan and/or yellow) in response to Cre-mediated recombination of the Brainbow cassette. The color-coding of neurons defined in the upper right box of A illustrates the various phenotypes possible in this injection paradigm. Neurons only infected with PRV-267 are marked by the VP26-mRFP reporter, a capsid surface fusion protein that is differentially concentrated in the cell nucleus (B) but produces punctate labeling in the cytoplasm (C) as capsids migrate out of the nucleus to be incorporated into mature virions. Selective infection with PRV-263 in the absence of Cre results in default expression of the dTomato reporter and homogeneous cytoplasmic labeling (D & E). Cre-mediated recombination of the Brainbow cassette in neurons replicating both PRV-263 and PRV-267 results in VP26-mRFP capsid labeling and cytoplasmic labeling by the cyan and/or yellow reporters (white arrows in F–H). Neurons infected by transneuronal passage of virus containing recombined genomes (*PRV-263re*) from dual infected neurons only express the cyan and/or yellow cytoplasmic reporters (I). Figure B is from IML of thoracic spinal cord, figures C & H are from raphe pallidus, figures D–H are from VMM. Marker bars in B, C, F, and G = 20 µm and those in D, E, H, and I = 25 µm.

The design of the experiment is illustrated in Figure 2A. Eight animals were included in the study. Animals were injected in pairs on different days and fresh aliquots of virus from the same viral stock were thawed for each pair of injections. Animals were deeply anesthetized using isoflurane and each kidney was exposed by a retroperitoneal approach. A total of 2 µl of PRV-263 was injected into the parenchyma of the left kidney (4 injections of 0.5 µl per site) using a 10 µl Hamilton syringe. An equivalent volume of PRV-267 was injected into the right kidney using the same procedure. We made an effort to standardize injections between animals by injecting at four similar sites along the greater curvature of each kidney and by marking the needle of the Hamilton syringe so that it penetrated the kidney parenchyma approximately 4 mm at each injection site. After injection, surgical incisions were sutured closed and animals received a subcutaneous injection of analgesic (Ketofen; 2 mg/kg). Upon full recovery from anesthesia animals were returned to their home cages in the BSL 2 laboratory where they lived for the balance of the experiment. The purified stocks of the viruses had concentrations of 3.4×10^8^ pfu/ml (PRV-263) and 5×10^8^ pfu/ml (PRV-267).

### Tissue Preparation

Animals deeply anesthetized with sodium pentobarbital were perfused transcardially with paraformaldehyde-lysine-periodate fixative [25] four (n = 2) and five (n = 6) days post inoculation. Aldehyde fixed tissues were postfixed and cryoprotected. The brain was sectioned with a freezing microtome at 35 µm/section through its rostrocaudal extent. The spinal cord was divided into cervical, thoracic and lumbosacral divisions and sectioned horizontally at 40 µm/section. Tissue was stored in cryoprotectant [25] at −20°C prior to immunocytochemical analysis. Details of all of these procedures have been published [26].

### Immunoperoxidase localizations

The invasive profiles of the recombinants were first determined by immunoperoxidase localization of infected neurons. Coronal sections at a frequency of 210 µm through the brain and horizontal sections at a frequency of 16 µm through the spinal cord were processed from each case. Viral immunoreactivity was detected with a rabbit polyclonal antiserum (Rb133) generated against acetone-inactivated virus [27]. This antiserum recognizes epitopes on all virally encoded proteins and was used at a 1∶10,000 dilution in conjunction with affinity purified, biotinylated donkey anti-rabbit secondary antibody (1∶200; Jackson ImmunoReseach Laboratories, Inc.; West Grove, PA) and Vectastain *Elite* avidin-biotin reagents (9 µl of each reagent combined 90 minutes before tissue incubation; Vector Laboratories; Burlingame, CA). Diaminobenzidine (DAB) was used as a substrate for the immunoperoxidase reaction; tissue was incubated in the DAB solution for 10 minutes prior to addition of 35 µl of H_2_O_2_/100 ml DAB solution to catalyze the reaction, and the reaction was terminated 3 minutes after H_2_O_2_ addition by repeated rinses in sodium phosphate buffer. Processed sections were mounted on Superfrost Plus microscope slides (Fisher Scientific, Pittsburgh, PA), dehydrated in graded alcohols, cleared in xylenes, and coverslipped with Cytoseal 60 (Richard-Allan Scientific, Kalamazoo, MI). The details of these procedures as applied in our laboratories have been published [26].

### Fluorescence microscopy

Sections of brain and spinal cord adjacent to those used for the immunoperoxidase analysis were analyzed using fluorescence microscopy. Sections were mounted on gelatin-coated slides, air dried, and coverslipped using Vectashield Hard Set mounting medium (Vector Laboratories, Burlingame, CA). The fluorophor profile of infected neurons was determined using an Olympus BX51 epifluorescence microscope equipped with filters specific for reporter proteins encoded by the dTomato, mCerulean, and EYFP genes as described previously [18]. Digital micrographs of each region were captured with a Hamamatsu camera (Hamamatsu Photonics, Hamamatsu, Japan) and analyzed using the procedures detailed in the next section.

### Data analysis

**Figure 3:**
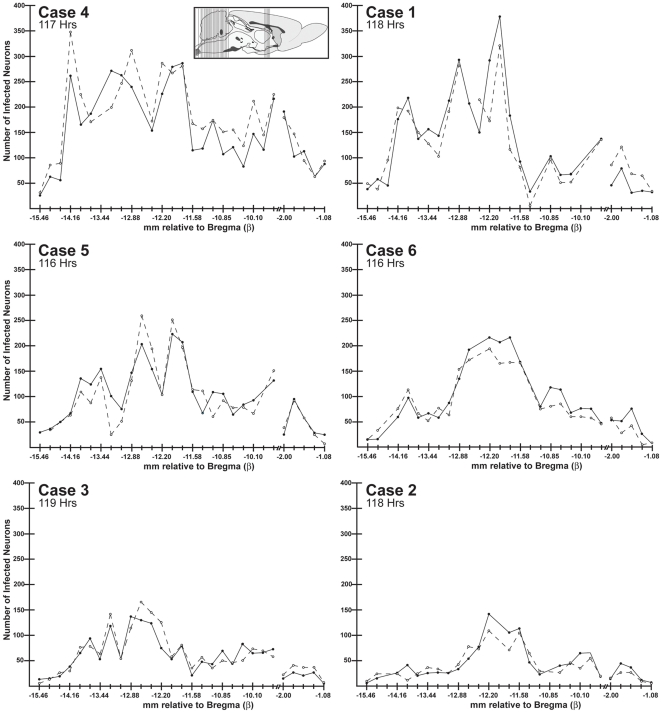
Neuroinvasive Profiles of PRV Recombinants. The number of infected neurons in 24 coronal planes sampling the neuraxis 5 days following injection of PRV 267 into right kidney (solid line) and PRV-263 into the left kidney (dashed line) is illustrated. Cases are arranged according to the magnitude of viral invasion. Case numbers are indicated in the upper left of each graph and the post inoculation survival interval is listed below each case number. The location of the planes of section sampled for each case is illustrated in the sagittal schematic included in the upper right of Case 4. The X-axis of each graph indicates the position of the 24 coronal planes relative to Bregma (β), an anatomical marker of the confluence of bone sutures on the rostral skull. The Y-axis indicates the number of infected neurons. Although the magnitude of infection varied between cases, the number of infected neurons on the left and right side of the brain was comparable for each coronal plane in each case. This finding is consistent with the conclusion that each recombinant invaded preautonomic circuitry from the kidney at equivalent rates in each experimental animal. The schematic diagram is adapted from the atlas Brain Maps: Structure of the Rat Brain [28].

We first characterized the extent of viral invasion of renal presympathetic circuits using immunoperoxidase localization of infected neurons in brain and spinal cord. The goals of this analysis were to determine if the invasiveness of each virus was equivalent and conformed to the distribution documented in our prior dual infection analysis of renal preautonomic circuitry [20]. To accomplish this we mapped the location of infected neurons in coronal sections through selected coronal planes through the neuraxis using StereoInvestigator image analysis software (version 8; Microbrightfield, Williston, VT). We selected 24 coronal sections that thoroughly sampled the renal preautonomic network across a 6.23 mm portion of the brain stem and five sections through a 0.92 mm portion of diencephalon that contained the PVN (Figure 3). Care was taken to encode the laterality of sections (*e.g.*, left and right) to ensure accurate recording of viral invasion of neural circuits innervating the left (PRV-263) and right (PRV-267) kidneys. Similarly, we matched the rostrocaudal levels of sections to ensure an accurate comparison of viral labeling between cases. These maps allowed a quantitative comparison of the neuroinvasiveness of each virus from the injected kidney (Figure 3) and also revealed the pattern of viral spread through the preautonomic network. To illustrate these maps we faithfully transferred labels of individual infected neurons to templates from the Brain Maps: Structure of the Rat Brain compiled by Swanson [28].

With these quantitative data in hand we then documented the fluorescence profile of infected neurons in the thoracic spinal cord, ventromedial medulla (VMM), rostroventrolateral medulla (RVLM), locus coeruleus (LC), and the paraventricular hypothalamic nucleus (PVN). These regions were selected for analysis because prior investigations demonstrated that they would contain neurons synaptically dedicated to the ipsilateral injected kidney as well as neurons that collateralize to circuits linked to both kidneys. Comparable coronal sections through these regions in each animal were examined and photographed. The fluorophor profile of all photographed neurons was determined using Adobe Photoshop software; fluorescence emitted by the protein products of the dTomato, mCerulean and EYFP genes was determined by examining color channels selective for each fluorophor. In this manner it was possible to determine with certainty the fluorophors expressed by each infected neuron.

## Results

Our experimental design takes advantage of a well characterized dual infection paradigm that results in predictable retrograde transneuronal passage of PRV recombinants from the kidneys [20]. The pattern of infection and the distribution of collateralized neurons observed in the present analysis recapitulated the findings documented in that foundational study, which used PRV recombinants expressing unique reporters (PRV-152; EGFP and PRV-BaBlu; β galactosidase) injected into separate kidneys. The predictable pattern of infection produced in this model system provided a strong foundation for the proof-of-principle observations reported in the following sections.

### Invasive Profiles of PRV Recombinants

There is a finite time period after initial infection of a neuron by PRV (about 6 hours) when the cell is permissive to infection by a second strain of PRV [29], [30]. Accordingly, we first determined the invasive profiles of PRV-263 and PRV-267 by conducting a quantitative analysis of the spread of each recombinant through the preautonomic network in dual injected animals. We localized infected neurons using immunoperoxidase procedures and obtained counts of neurons on each side of the brain using an image analysis system. Immunoperoxidase localization of viral antigens does not distinguish between the recombinants infecting individual neurons but does provide an informed evaluation of the extent of spread of each recombinant through the predominantly lateralized circuitry synaptically linked to each kidney.

The temporal kinetics and pattern of invasion of renal preautonomic circuitry for PRV-263 and PRV-267 recapitulates that documented in our prior studies. Replication and transneuronal passage of each recombinant produced infection of sympathetic preganglionic neurons in the intermediolateral cell column (IML) of thoracic spinal cord and subsequent retrograde transneuronal passage through synaptically connected neurons in the renal preautonomic network. At four days after kidney infection, PRV immunopositive neurons were largely confined to areas in the brain stem that give rise to direct descending projections to the thoracic spinal cord (*e.g.*, RVLM and A5; data not shown). One day later, the number of infected neurons in regions infected at 4 days increased and the infection spread transneuronally to neurons in other regions of the medulla, midbrain and forebrain (Figure 2A). In every case, the distribution of infected neurons conformed to that previously documented in prior viral transneuronal tracing studies. Importantly, the absence of infected neurons in the dorsal motor nucleus of the vagus (a parasympathetic cell group innervating the visceral) demonstrated that organ-specific transport of virus was not compromised by leakage of viral inoculum into the peritoneal cavity.

**Figure 4:**
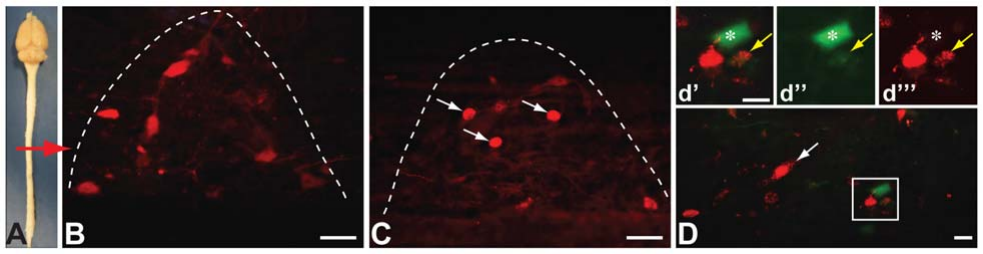
Fluorphor Expression in Spinal Cord IML. Infected SPN neurons in the thoracic IML after injection of PRV recombinants into the kidneys are illustrated. The approximate level of thoracic spinal cord illustrated in figures B–D is designated by the red arrow on the dissection of brain and spinal cord shown in figure A. Figure B & C illustrate infected SPNs in IML segments ipsilateral to kidneys injected with PRV-263 (B) or PRV-267 (C) in case 4. The default dTomato reporter fills the soma and proximal dendrites of SPNs infected only with PRV-263 while capsids tagged with the VP26-mRFP fusion protein densely label the nucleus (white arrows) of neurons only infected with PRV-267. Figure D illustrates the fluorophor profiles of infected SPNs in an IML segment ipsilateral to the kidney injected with PRV-267 (case 1). Labeled capsids are concentrated in the nuclei of infected SPNs but are also apparent in the cell cytoplasm (white arrow). In addition, IML neurons in this segment express cytoplasmic reporters of the recombined Brainbow cassette, either alone or with the VP26-mRFP reporter. The yellow arrows in d′–d″ illustrate a dual infected neuron expressing mRFP and cytoplasmic reporters of the recombined Brainbow cassette. The neuron labeled with the asterisk in d′ & d‴ expresses cytoplasmic reporters of the recombined Brainbow cassette, but no mRFP labeled capsids, and was infected by retrograde transneuronal passage of virus from dual infected neurons. Marker bars for figures B & C = 50 µm; marker bars for D and d′ = 20 µm. The magnification is equivalent for d′–d‴.

**Figure 5:**
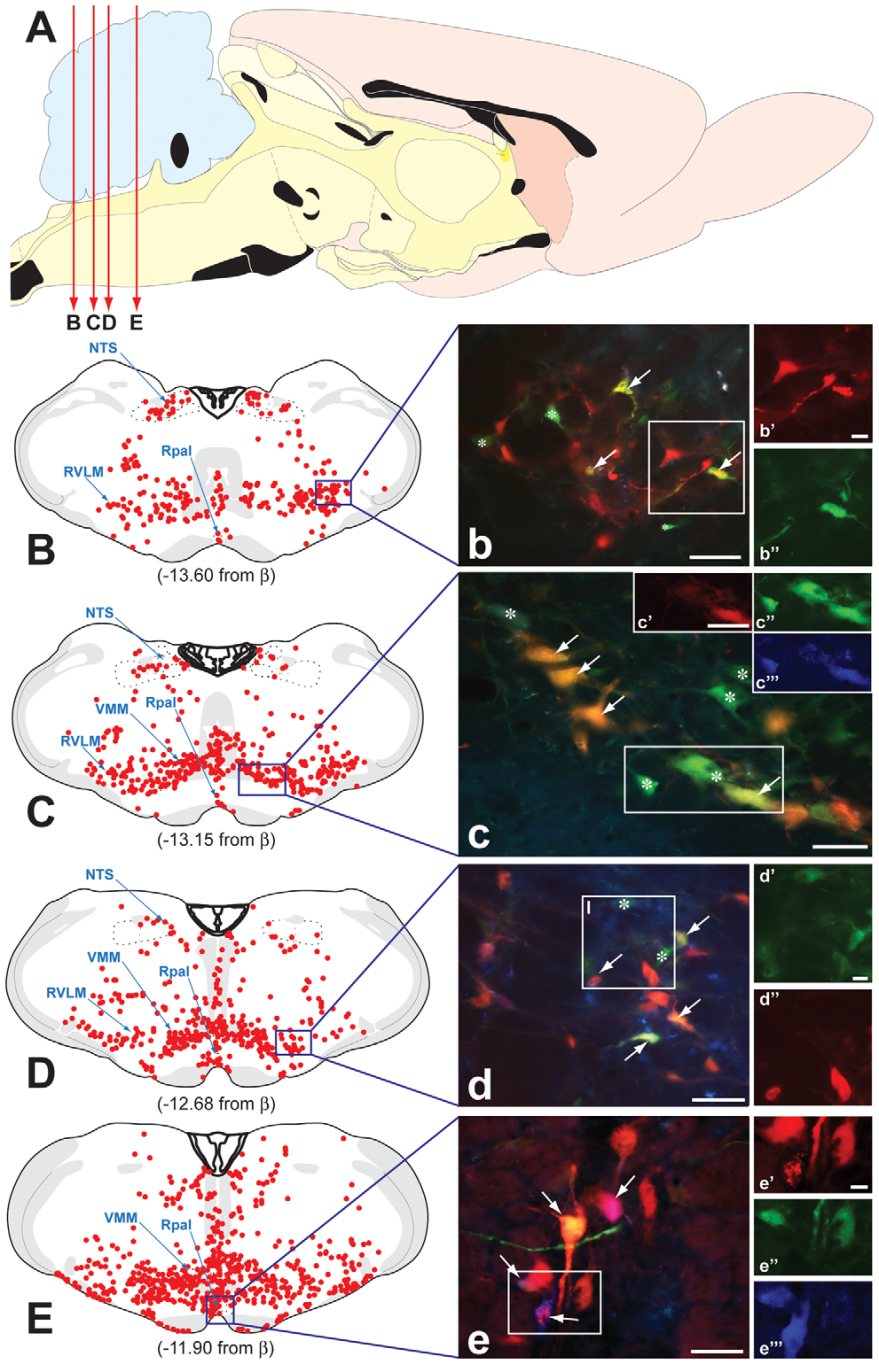
Fluorphor Expression in Caudal Brainstem. The distribution of infected neurons throughout the caudal brainstem is illustrated. The red lines described in figure A illustrate the location of the coronal planes illustrated in figures B through E. Figures B through E map the distribution of infected neurons detected by immunoperoxidase localization of viral antigens in case 1. Each red dot indicates the position of an infected neuron and the position of each section with respect to Bregma (β) is indicated below each map. Figures b through e illustrate the fluorescent profiles of neurons in the RVLM (b & d), VMM (c) and raphe pallidus (Rpal; e) in sections adjacent to those mapped for immunoperoxidase localization of viral antigens. The relative position of each field illustrated in figures b through e is illustrated in the boxed area of figures B through E. The fluorescence in figures b through e is a composite of that revealed by the filters specific for dTomato, mCerulean and EYFP. The fluorescence signal from individual color channels in the boxed areas of figures b through e is shown at higher magnification in adjacent photomicrographs and insets (lower case letters marked apostrophes). White arrows mark dual infected neurons expressing VP-mRFP labeled capsids and EYFP and/or mCerulean reporters of the recombined Brainbow cassette. Cells marked by the asterisks are expressing reporters of the recombined Brainbow cassette but do not contain VP26-mRFP labeled capsids. The absence of labeled capsids in these cells indicates that they were infected by transneuronal passage of virus from a dual infected neuron. The photomicrographs illustrated in b–d are from case 1 and that shown in figure e (and at higher magnification in figure 2H) is from case 3. The schematic diagrams are adapted from the atlas Brain Maps: Structure of the Rat Brain [28]. NTS = nucleus of the solitary tract; RVLM = rostroventrolateral medulla; VMM = ventromedial medulla. Marker bars for b–e and c′ = 50 µm; marker bars for b′, d′, and e′ = 10 µm.

**Figure 6:**
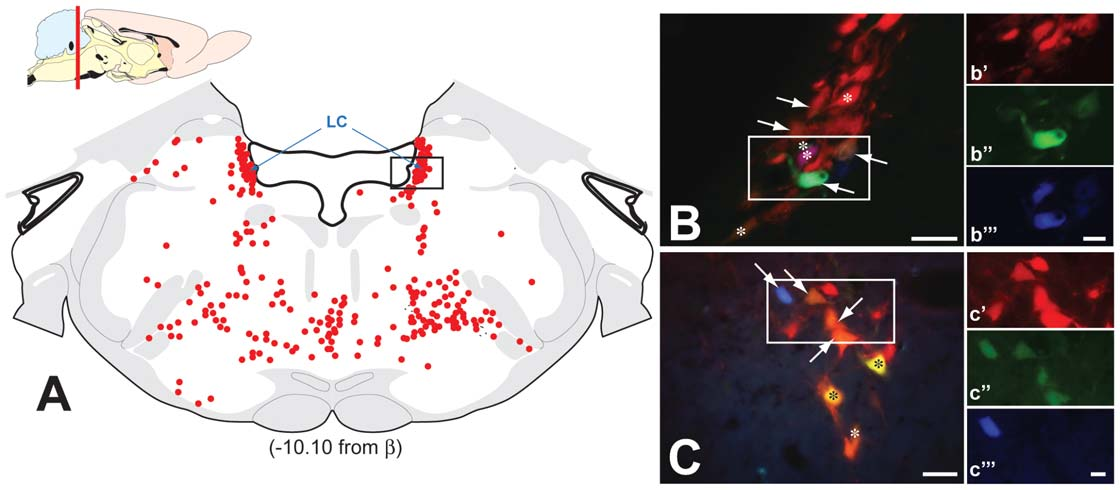
Fluorphor Expression in Rostral Brainstem and LC. The distribution of infected neurons in rostral brainstem at the level of the locus coeruleus (LC) is illustrated. The schematic sagittal section in the upper left of figure A illustrates the position of the plane sampled in the rostrocaudal axis of the brain. The coronal schematic in figure A maps the distribution of infected neurons detected by immunoperoxidase localization of viral antigens in case 1. Each red dot indicates the position of an infected neuron in a section −10.10 mm relative to Bregma. Figures B and C, also from case 1, illustrate the fluorescent profiles of LC neurons in sections adjacent to that shown in A. The box in A marks the relative position of images B and C, which illustrate reporter fluorescence revealed by the filters specific for dTomato, mCerulean and EYFP. Fluorescence signal in the boxed areas of B & C is shown at higher magnification in b′–b‴ and c′–c‴. White arrows in B & C indicate neurons dual infected neurons. Cells marked by the asterisks are expressing reporters of the recombined Brainbow cassette but do not contain VP26-mRFP labeled capsids. The schematic diagram is adapted from the atlas Brain Maps: Structure of the Rat Brain [28]. Marker bars in B & C = 50 µm; marker bars in b‴ and c‴ = 20 µm.

**Figure 7:**
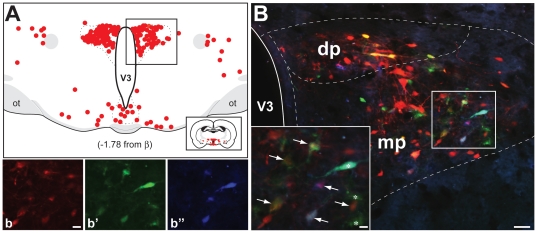
Fluorophor Expression in Diencephalon and PVN. The distribution of infected neurons in diencephalon at the level of the paraventricular hypothalamic nucleus (PVN) is illustrated. Figure A maps infected neurons detected by immunoperoxidase localization of viral antigens in case 1. Each red dot indicates the position of an infected neuron and the position of the section with respect to Bregma (β) is indicated below the schematic. Figure B illustrates the fluorescent profiles of neurons in parvicellular PVN subdivisions in a section adjacent to that shown in A. The fluorescence in figure B is a composite of that revealed by the filters specific for dTomato, mCerulean and EYFP and the boxed area is shown at higher magnification in the inset. Figures b–b″ show the fluorescence for individual channels in the same area as the inset in figure B. White arrows mark dual infected neurons and asterisks mark neurons that only express reporters of the recombined Brainbow cassette. The schematic diagram is adapted from the atlas Brain Maps: Structure of the Rat Brain [28]. Marker bar in figure B = 50 µm and the marker bar in the inset of figure B = 10 µm. Figures b–b″ are of the same magnification and the marker bar in b = 20 µm.

Figure 3 illustrates the number of infected neurons on the left and right side of the brain in multiple coronal planes sampling the renal preautonomic network in the six cases processed 5 days after kidney injection. The number of infected neurons on the left and right sides of the brain were remarkable consistent in each animal, indicating that both PRV-267 and PRV-263 invaded the preautonomic network at similar rates and extents from each kidney. Additionally, each recombinant infected the same cell groups (*e.g.*, Figures 4, 5, 6, 7).

Interestingly, the extent to which the preautonomic network was infected varied to a considerable extent between animals surviving five days. The post inoculation survival interval for these animals ranged from 116 to 119 hours and the animals were all injected and perfused midway through the light phase of the circadian cycle (between 11 am and 3 pm). All injections were made from the same stock of virus of constant titer and a freshly thawed aliquot of virus was used to inject each pair of animals. Similar variations in the magnitude of infection between animals were noted in the study by Cano et al. [20] employing PRV-152 (EGFP reporter) and PRV-BaBlu (β galactosidase reporter) in this model. Such variations may be the result of differences in the density of sympathetic innervation of kidney or to variations in the number of infect cells within the kidney that become infected and amplify virus available for uptake and retrograde transport by sympathetic afferents, possibly due to differences in immune response to infection. In any event, the quantitative analysis in this study provided strong evidence that the invasion of preautonomic circuits from each kidney was equivalent for each recombinant within an animal, thereby optimizing the probability of achieving dual infection of collateralized neurons.

### Fluorescence profiles of infected neurons

Neurons infected by PRV-267 express a red punctate signal that produces a dense labeling of cell nuclei early in viral replication (Figures 2B) followed by the appearance of red puncta in the cytoplasm later in infection (Figures 2C). This labeling is predicted by the demonstrated sequence of capsid assembly (nuclear) and envelopment (cytoplasmic) characteristic of viral replication and spread [31]. The expression profiles of fluorescent reporters resulting from infection of neurons with PRV-263, either alone or in the presence of Cre, have been characterized [17], [18]. In the absence of Cre, the cytoplasm of infected cells fills with the dTomato (red) reporter expressed from the Brainbow cassette (Figures 2D and E). However, in the presence of Cre the red reporter gene is excised resulting in expression of either the yellow (EYFP) or cyan (mCerulean) reporters (Figures 2F–I).

Dual infected neurons in the present study were marked by the punctate VP26-mRFP capsid marker of PRV-267 and the reporters of the recombined Brainbow cassette (white arrows in Figures 2F–H). Once Cre acts, no more recombination is possible thus rendering the expression characteristics of the resulting progeny virus permanent. Transneuronal passage of recombined PRV-263 will therefore produce either cytoplasmic cyan or yellow reporters. Whether transneuronally infected neurons express multiple cytoplasmic reporters of PRV-263 infection and also contain VP26-mRFP appears to depend upon the mixture of progeny virus that passes transneuronally from dual infected neurons (see Discussion for supporting literature). However, when the cytoplasmic reporters of the recombined Brainbow cassette are present in cells lacking punctate VP26-mRFP labeling (Figure 2I) the neurons should have been infected by retrograde transneuronal passage of virus from one or more dual infected neurons.

### Reporter gene expression in neurons replicating only one recombinant

Reporter gene expression in sympathetic preganglionic neurons (SPNs) of thoracic spinal cord illustrated the distinctive reporters of neuronal infection with PRV-263 or PRV-267. Infection of SPNs in the IML revealed the largely lateralized sympathetic outflow to the kidneys and other autonomic targets; *e.g.*, SPNs innervating the kidney injected with PRV-263 displayed homogeneous expression of the dTomato reporter throughout the somatodendritic compartment (Figure 4A) while SPNs infected by retrograde transport of virus from the kidney injected with PRV-267 contained dense concentrations of mRFP puncta in cell nuclei (Figure 4B).

The largely lateralized organization of preautonomic circuitry innervating SPN outflow to each kidney predicts that the majority of neurons infected with only one virus will be concentrated on the side of the brain ipsilateral to the injected kidney. Examination of each group of infected neurons contributing to the preautonomic circuitry on the left and right sides of the brain confirmed this prediction. The majority of infected neurons in each node within the preautonomic network infected with only one virus displayed fluorescence profiles consistent with the genotype of virus injected into the ipsilateral kidney.

### Reporter gene expression in dual infected neurons

We analyzed regions of the spinal cord, medulla and diencephalon previously shown to contain neurons infected through collateralized axonal projections to neural circuits innervating each kidney [20]. If neurons infected with PRV-267 are producing biologically active Cre then we should observe dual infected neurons expressing cyan and/or yellow reporters. Similarly, the presence or absence of the unique reporter of PRV-267 infection (punctate VP26-mRFP) in neurons expressing cyan and yellow fluorescence should allow us to discriminate neurons replicating both viruses from synaptically connected neurons infected by transneuronal passage of virus from dual infected neurons. We selected the thoracic spinal cord, RVLM, VMM, LC and PVN for this analysis because of the well-known collateralized connections of neurons in these cell groups within the renal preautonomic network.

As noted above, we observed large numbers of infected SPNs in the thoracic spinal cord that were marked by only PRV-267 or PRV-263 and were concentrated in IML segments ipsilateral to the injected kidney (Figure 4). However, as reported previously [20], we also observed interneurons within IML segments that were infected by retrograde transneuronal passage of virus from the contralateral IML. These neurons were most prevalent in cases with the most extensive transport through the preautonomic network and contained punctate VP26-mRFP labeling in the nucleus and cytoplasm as well as yellow and blue cytoplasmic reporters of the recombined Brainbow cassette (yellow arrows in Figures 4d′–4d″). This pattern of reporter gene expression is consistent with dual infection of neurons by PRV-263 and PRV-267 through collateralized axons that synapse upon SPNs bilaterally in thoracic spinal cord.

The profile of gene expression in RVLM neurons is predicted by the known connectivity of the RVLM, which is characterized by a large projection to the ipsilateral IML and lesser projections to the contralateral RVLM and IML. Data consistent with this prediction are shown in Figures 5B and D. We observed RVLM neurons expressing only the cytoplasmic dTomato reporter (PRV-263 infection), neurons only expressing the punctate nuclear VP26-mRFP reporter (PRV-267 infection), and neurons expressing the conditional cytoplasmic reporters (cyan and/or yellow) in combination with the punctate VP26-mRFP labeling of nuclei (dual infected neurons) (Figures 5b and d). The largest proportion of neurons expressing these phenotypes was concentrated in the rostral aspect of RVLM, which is the portion of this cell group that gives rise to the largest portion of the reticulospinal projection to thoracic cord.

Reporter gene expression in VMM reflected documented descending reticulospinal projections to thoracic cord, reciprocity of connections to other nodes within the renal preautonomic network (e.g., RVLM), and local circuit connections within the VMM (Figure 5C). Direct descending projections to the thoracic cord were marked by cytoplasmic localization of the dTomato reporter or punctate VP26-mRFP labeling in neurons replicating only one virus, with the largest proportions of each of these groups present on the side of the brain ipsilateral to the injected PRV recombinant. We also observed neurons that contained both markers and were therefore infected by collateralization of axons to efferent pathways synaptically linked to both kidneys (white arrows in Figure 5c). Single and dual infected neurons were present in all subdivisions of the VMM, including raphe pallidus, but were most prevalent in the rostral third of this cell column, with the highest concentration occurring in the areas immediately lateral to the pyramids (Figure 5C and E; 5c and e).

Retrograde transneuronal infection of the LC also produced a pattern of infected neurons that conformed to that previously documented after injection of virus into the kidney [20]. The cases that displayed more limited invasion of preautonomic circuits (*e.g.*, animals surviving 4 days and cases 2 and 3 from the 5 day survival group) exhibited neurons largely confined to the ventral third of the LC. The majority of infected neurons in a single cell group were infected by the recombinant injected into the ipsilateral kidney, but a subset was infected by both recombinants. In cases with the most extensive transport of virus through preautonomic circuitry, infected neurons were observed throughout the dorsoventral extent of the LC bilaterally (Figure 6A). However, neurons replicating both recombinants remained confined to the ventral third of the LC (white arrows in Figures 6B and C). These finding confirm and extend those reported by Cano and colleagues following injection of PRV-152 (EGFP reporter) and PRV-BaBlu (β-galactosidase reporter) into separate kidneys.

Retrograde transneuronal infection of neurons in the PVN occurred in the parvicellular subdivisions of this diencephalic cell group. Only scattered infected neurons were found in the PVN in animals analyzed four days following injection of virus into the kidneys. Five days following kidney injection we observed numerous infected neurons in the dorsal, medial and posterior parvicellular subfields (Figure 7A). Figure 7B illustrates the distribution and phenotype of neurons typically observed in animals exhibiting the most robust infection of preautonomic circuitry (*e.g.*, cases 1 and 4). In each case, infected neurons were present within in both the dorsal and medial parvicellular subfields of PVN. Dual infected neurons (*e.g.*, white arrows in 7b) were a subset of a larger population infected only with the recombinant injected into the ipsilateral kidney.

### Transneuronal infection from dual infected neurons

The expression of conditional reporters of the Brainbow cassette throughout the preautonomic network, while confirming the presence of collateralized neurons, also revealed new insights into the synaptic organization of preautonomic synaptology. For example, we observed infected neurons that replicated the recombined Brainbow cassette (expressing cyan and yellow cytoplasmic reporters), but did not express punctate VP26-mRFP labeling. Since the presence of punctate VP26-mRFP marks cells infected with PRV-267, neurons that exclusively express cytoplasmic reporters of the recombined Brainbow cassette should have been infected by virtue of their synaptic linkage to dual infected neurons (*i.e.*, are presynaptic to dual infected neurons). This synaptic relationship cannot be distinguished in dual infection approaches that do not involve conditional reporter expression (*e.g.*, injection of PRV152 & PRV-BaBlu). Neurons of this phenotype (marked by asterisks in Figures 4, 5, 6, 7) were observed in each of the cell groups analyzed in this study and their prevalence appeared to vary among cell groups. For example, neurons displaying this phenotype were prevalent within RVLM, VMM and LC but were rarely observed within raphe pallidus. A more detailed analysis incorporating a larger sample size and quantitative analysis is necessary to determine the relative proportions of these neurons within individual cell groups of the renal preautonomic network. Nevertheless, the ability to discriminate these neurons from dual infected cell groups provides another level of insight into the synaptology of neural networks identified in dual infection paradigms.

## Discussion

The findings reported in this manuscript document a new viral transneuronal tracing approach that can be used to identify connections to neurons within a complex network whose axons collateralize to influence separate targets. To test the utility of this approach, we used a well documented dual infection animal model in which PRV recombinants that express unique reporters are injected into separate kidneys [20]. The use of PRV-263 and PRV-267 in this model system provides unique insights into the synaptic organization of complex circuits that cannot be resolved in dual infection studies employing isogenic PRV recombinants that constitutively express unique reporters (*e.g.*, PRV-152 & PRV-BaBlu). Particularly important in this regard is the ability to discriminate neurons presynaptic to dual infected neurons. Nevertheless, it is important to emphasize that the method does not permit a definitive identification of all neurons providing synaptic input to dual infected collateralized neurons and thereby provides a qualitative rather than quantitative approach for identifying these neurons.

The method builds upon recent studies in which we reported the construction and characterization of PRV-263 [17] and demonstrated the ability of lentivirus mediated Cre expression to produce conditional reporter expression from a Brainbow cassette [19] carried by PRV-263 in targeted populations of neurons [18]. Here we describe the construction and use of PRV-267, which serves both as a transneuronal tracer and a vector for circuit related expression of Cre. To our knowledge this is the first demonstration of the ability to deliver biologically active Cre in a circuit specific fashion across multiple synapses. The fact that Cre is expressed throughout the polysynaptic circuit infected by PRV-267 is validated both by the pattern and kinetics of conditional reporter expression observed within the CNS following separate injections of PRV-267 and PRV-263 into the kidneys. Importantly, the present data confirm the identity and organization of neurons within the preautonomic network previously shown to collateralize to regulate both kidneys [20]. Considered with evidence that recombination of the Brainbow cassette only occurs in the presence of Cre, this is an important confirmation that PRV-267 is producing biologically active Cre in the neurons that it infects.

Importantly, the insights derived from the use of PRV-263 and PRV-267 in dual infection experiments are not limited to the ability to identify neurons that collateralize to influence separate targets. We observed neurons that expressed reporters of the recombined Brainbow cassette but not the unique reporter of PRV-267 infection (punctate VP26-mRFP). These neurons can only have been infected subsequent to Cre mediated recombination and transneuronal passage of the PRV-263 genome. Using *in vitro* analysis Kobiler and colleagues demonstrated that Cre-mediated recombination occurs prior to replication of PRV-263 and that a remarkably small number of viral genomes – as few as seven – are expressed, replicated and assembled into virions [17]. This interesting bottleneck may limit the population of virions that can spread transneuronally and express their genomes. In any case, even if PRV-263 and PRV-267 co-infect a single neuron, the data of Kobiler and colleagues indicates that the probability of second- and third-order neurons being infected by both recombinants drops after each transneuronal passage. Therefore, neurons displaying only cytoplasmic reporters of the recombined PRV-263 genome, and no reporters of PRV-267 infection (punctate VP26-RFP), likely represent neurons that were infected from the early transneuronal passage of progeny virus containing the recombined PRV-263 genome from a dual infected neuron. Similarly, early transneuronal passage of PRV-267, and not PRV-263 recombinants, from dual infected neurons would produce neurons only expressing the PRV-267 genome that are indistinguishable from neurons connected only to the PRV-267 infected kidney. Thus, data derived from this approach must be interpreted conservatively and conclusions on the synaptology of the circuit based only upon positive unequivocal results. In this regard, the singular expression of PRV-263 reporters of the recombined Brainbow cassette provides an unambiguous identification of neurons presynaptic to dual infected neurons.

As noted above, the *in vitro* data of Kobiler and colleagues demonstrated that Cre-mediated recombination of the PRV-263 genome occurs prior to replication of the virus. However, there is a chance that several incoming PRV-263 genomes will initiate replication before recombination can occur, even in the presence of PRV-267. This can result in neurons that were infected with both viruses expressing the default dTomato reporter along with the reporters liberated by Cre mediated recombination. Under these circumstances it is possible that a single dual infected neuron can replicate up to four different viral genomes (PRV-267, PRV-263red, PRV-263yellow, and PRV-263blue) and transneuronal infection of synaptically connected neurons would sample any combination of these replicated genomes. Indeed, we often observed neurons *in vivo* that expressed dTomato (a reporter of the uncombined PRV-263 genome) along with the mCerulean and EYFP reporters of recombination.

The functional implications of being able to identify neurons presynaptic to collateralized neurons are apparent in our data. Jansen and colleagues previously documented neurons within the preautonomic network that were co-infected by retrograde transneuronal transport of recombinant strains of PRV from the adrenal gland and superior cervical ganglion as a means of identifying “command” neurons instrumental in the initiation of the “fight-or-flight” response to stressful stimuli [32]. The neurons identified in their investigation are among the dual infected neurons observed in our investigation and include areas that have been identified as important mediators of neural responses stress. The LC is among the regions identified in our analysis that were not included in the “command” neurons identified by Loewy and colleagues. Nevertheless, the LC is prominent among the cell groups activated by stressful stimuli and it has been postulated to play a prominent role in orchestrating behavioral and physiological responses to stressors [33], [34], [35]. Importantly, available evidence indicates that the LC does not exert its influence upon sympathetic outflow through direct reticulospinal projections to SPNs in the IML [36]. Rather, LC neurons project to components of the preautonomic network that, in turn, project directly to SPNs (e.g., RVLM & VMM) and also influence sympathetic outflow indirectly through projections to regions that influence affect [37]. Our data suggest that the LC contains a large population of neurons presynaptic to dual labeled neurons, an observation consistent with a prominent role for the LC in the global activation of sympathetic outflow that is a cardinal feature of the fight-or-flight response. Our data are also consistent with a similar functional role for the hypothalamic PVN, which also contained prominent populations of neurons presynaptic to collateralized neurons. Definitive support for these hypotheses requires quantitative analysis of a larger sample size, but the possibility illustrates the potential power of the combined use of PRV-263 and PRV-267 in dual infection analysis of neural circuitry.

The ability to express Cre in a circuit related manner through PRV-267 infection and transneuronal passage also has other experimental applications. For example, PRV-267 can be used to mediate recombination of floxed genes in transgenic mice in a circuit-defined manner. Given the expanding list of floxed genes that are widely available (*e.g.*, see the list on the web site of Andras Nagy at the Samuel Lunenfeld Research Institute at Mount Sinai Hospital; http://www.mshri.on.ca/nagy/) this possibility markedly expands the utility of PRV-267 for functional studies in a variety of systems. Additionally, the virus can be used to produce circuit related conditional reporter expression in the nervous system of the Brainbow mouse [19].

In conclusion, we have described a new viral tracing method based on the polysynaptic tracing properties of PRV, the ability to express biologically active Cre from the PRV genome, and the conditional reporter capabilities of the Brainbow cassette. The method enables identification of neurons that collateralize within a complex network to exert regulatory control over distant separate targets. It provides a means of expressing Cre in a circuit specific fashion from a replication competent PRV recombinant (PRV-267) and relies upon Cre-dependent combinatorial expression of fluorescent reporters from a Brainbow cassette carried by second PRV recombinant (PRV-263). The unique reporter phenotypes produced in dual infection studies employing these recombinants provides unique insights into the synaptic organization and function of polysynaptic networks and increases the diversity of viral transneuronal tracing tools available for circuit analysis.
